# Evaluating the Utility and Implementation Barriers of a Liquid Biopsy Biomarker Test Early in the Lung Cancer Diagnostic Pathway to Improve Timeliness of Palliative Systemic Therapy

**DOI:** 10.3390/curroncol33010042

**Published:** 2026-01-13

**Authors:** Adi Kartolo, Laura Semenuk, Harriet Feilotter, Colleen Savage, Alexander Boag, Wilma Hopman, Geneviève Digby, Mihaela Mates

**Affiliations:** 1 Department of Oncology, McMaster University, Hamilton, ON L8S 4L8, Canada; 2 Escarpment Cancer Research Institute, Hamilton, ON L8S 4L8, Canada; 3 Molecular Genetics Laboratory, Kingston Health Sciences Centre, Kingston, ON K7L 2V7, Canada; 4 Department of Pathology and Molecular Medicine, Queen’s University, Kingston, ON K7L 3N6, Canada; 5 Department of Laboratory Medicine and Pathobiology, University of Toronto, Toronto, ON M5S 3K3, Canada; 6 Department of Oncology, Queen’s University, Kingston, ON K7L 3N6, Canadawilma.hopman@kingstonhsc.ca (W.H.); 7 Vice-Principal Research Office, Queen’s University, Kingston, ON K7L 3N6, Canada; 8 Division of Respirology and Sleep Medicine, Department of Medicine, Queen’s University, Kingston, ON K7L 2V7, Canada

**Keywords:** cfDNA, liquid biopsy, biomarker, lung cancer, systemic therapy, diagnostic timeliness

## Abstract

**Purpose:** Timeliness of systemic therapy initiation for advanced lung cancer is highly dependent on pathology and molecular pathology laboratory services. Here, we aimed to prospectively evaluate liquid biopsy as a potential strategy to expedite systemic therapy decision-making in lung cancer management. **Patients and Methods:** This prospective cohort study included consecutive patients with suspected lung cancer seen at the time of initial specialist consultation who underwent both liquid and solid tumour biopsy (group A) and patients with confirmed lung malignancy who underwent solid tumour biopsy alone (group B), between 1 February 2022 and 31 May 2023. Due to laboratory factors, liquid biopsies were processed in batches of 13, whereas solid tumour biopsies were processed individually upon receipt, as per standard practices. Co-primary endpoints included the time from solid versus liquid biopsies to biomarker reporting and palliative systemic therapy initiation. **Results:** A total of 324 patients were included in the study. The median time from date of blood draw to date of liquid biopsy result was 78 days. For group A (*n* = 50), the median time from date of solid tumour biopsy to biomarker reporting was 22 days, and the median time from date of solid tumour biopsy to palliative systemic therapy was 42 days. The median time from date of liquid biopsy blood draw to palliative systemic therapy initiation was 56 days. For group B (*n* = 274), the median times from date of biopsy to biomarker reporting and to palliative systemic therapy initiation in all patients were 22 and 47 days, respectively. **Conclusions:** While we did not demonstrate improvement in timeliness of biomarker reporting or systemic therapy initiation with liquid biopsy, several barriers leading to delay in liquid biopsy reporting were identified due to unexpected COVID-19-related supply chain disruption and the cost-limiting need to batch sample analysis. Further studies that address the identified barriers are warranted to assess the potential improvement in timeliness of care, should liquid biopsy analysis be implemented in real-time.

## Simple Summary

This study attempted to determine whether a blood test (known as a liquid biopsy) could help shorten the time for lung cancer patients to receive biomarker results and start treatment compared with a traditional solid tissue biopsy. We studied 324 patients undergoing evaluation for lung cancer and found that while the blood test was performed an average of 11 days earlier than the tissue biopsy, the test results took much longer to be reported (78 vs. 22 days). These delays were not due to the test itself, but rather due to laboratory factors, such as needing to batch the samples to run the analysis economically rather than analyzing them individually upon receipt, as well as factors related to pandemic supply shortages. Despite these barriers, our study also demonstrated good reliability of liquid biopsy in revealing the same biomarker results compared with the standard tissue biopsy, such that it would be important to further explore the utility of liquid biopsy in improving the timeliness of lung cancer care while addressing identified laboratory barriers.

## 1. Introduction

Systemic therapies for non-small-cell lung cancer (NSCLC) are increasingly guided by tumour biomarker results, such as protein expression markers (program death ligand–1 [PDL-1]) and molecular profiling [1,2,3]. Timeliness of systemic therapy initiation is, therefore, highly dependent on pathology and molecular pathology laboratory services. Given that lung cancer outcomes are heavily influenced by the timeliness of treatment initiation [4,5], strategies that have the potential to expedite decision-making regarding systemic therapies could improve patient outcomes.

One strategy that has demonstrated potential for expediting the timeliness of treatment is the use of liquid biopsy (or circulating tumour DNA [ctDNA]) to identify actionable genomic biomarkers. These ‘actionable mutations’ are defined as tumour DNA changes for which a specific targeted systemic therapy is available to treat the patient. DNA fragments released from apoptotic or necrotic primary tumour cells can be identified by analysis of DNA derived from a simple blood draw [6]. Previous studies demonstrated high concordance for clinically relevant mutations between ctDNA and primary tumour DNA in patients with NSCLC [6,7,8].

Similar to many other Canadian institutions, our local practice at the Kingston Health Sciences Centre (KHSC) is to send tissue samples from patients with suspected lung cancer to the pathology lab for confirmation of the histologic diagnosis and for analysis of PD-L1 by immunohistochemistry. In the setting of a non-squamous histology, a series of cut sections is transferred to the molecular laboratory for analysis of critical molecular biomarkers. The molecular laboratory uses a massively parallel sequencing (MPS) approach to determine the mutational status of several relevant molecular targets, a process that can take up to 2 weeks, with the total time from the tissue biopsy acquisition to molecular results being upwards of 4 weeks. The consequence to patients is a substantial delay from lung cancer diagnosis to first systemic therapy, with baseline data from our region in 2016 demonstrating an average of 38 days from diagnosis to systemic therapy initiation [9]. A previous Canadian study reported that only 21% of patients with advanced NSCLC had biomarker results available at their initial oncology consultation, which resulted in an additional 29-day wait time between initial consultations and treatment start date [10]. Meanwhile, not all tissue biopsy samples are able to be processed for biomarkers due to sample size or quality, while other patients may not be candidates for biopsy due to patient comorbidities, technical factors, and/or procedural limitations or availability [11]. As such, there is significant room for improvement in the timeliness of treatment, quality of care, and accessibility of treatment options for patients with lung cancer. The emerging literature highlights promising aspects of liquid biopsy assays to complement conventional tissue sampling in addressing these barriers to care, but limitations regarding their sensitivity and specificity remain, and their utility as a diagnostic tool requires further evaluation [12].

In this study, we aimed to prospectively evaluate the potential impact of incorporating a liquid biopsy molecular analysis early in the diagnostic pathway for patients with suspected lung cancer by evaluating the impact on timeliness of biomarker reporting and time to systemic therapy initiation. Our co-primary study objectives were to describe the time from solid versus liquid biopsies to biomarker reporting, as well as the time from solid versus liquid biopsies to palliative systemic therapy initiation. Secondary objectives were to assess the timeliness of various points in care through the entire cancer journey, from initial clinical assessment to palliative systemic therapy initiation.

## 2. Methodology

### 2.1. Local Context

This was a prospective cohort study that took place at KHSC, an academic tertiary care centre that serves a catchment area of more than 500,000 people in Southeastern Ontario, Canada, who are covered with universal healthcare. In this region, the Lung Diagnostic Assessment Program (LDAP) is a rapid assessment clinic and primary clinical pathway for the evaluation of patients with imaging findings suspicious for lung cancer, resulting in more than 50% of patients in the region being diagnosed with lung cancer through the LDAP. This program is primarily led by respirologists who facilitate diagnostic and staging evaluations for patients and coordinate subsequent multidisciplinary assessment with oncologists.

### 2.2. Study Work Flow

**Figure 1:**
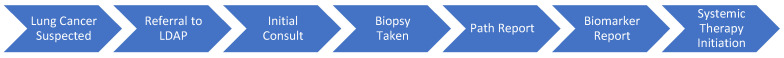
Therapeutic flow chart.

We offered liquid biopsies to patients seen in the LDAP at initial consultation for imaging findings suspicious for clinical stage III or IV lung cancer. Consenting patients underwent a blood draw collection of 40 mm of peripheral blood into Streck tubes, which were sent to the molecular diagnostic laboratory for ctDNA extraction, quality control assessment, and Massive Parallel Sequencing (MPS) analysis. During the same LDAP clinic visit, physicians coordinated a solid tumour biopsy procedure in accordance with standard clinical practices and regional procedural availability. Solid tumour biopsies were analyzed as per standard practices, while liquid biopsies were analyzed using a small panel that interrogated DNA markers only. Where there was a discordance between molecular markers identified in liquid and solid biopsies, we used information derived from the solid biopsy for final treatment decisions, as per current clinical guidelines. Figure 1 depicts our overall workflow.

For the purposes of this study, actionable mutations were defined as DNA changes for which a specific targeted systemic therapy was available to treat the patient, and they included EGFR exon 19 deletion, exon 20 insertion, exon 21 L858R, BRAF V600E/K, and KRAS G12C. ALK-EML5 and MET exon 14 skipping were not included, as these fusion alterations were not detectable by the ctDNA MPS assay, which was used in this study. Details of ctDNA processing are described below in Section 2.5 (MPS Assay).

### 2.3. Study Participants

In the analysis, we included all patients undergoing evaluation for suspected lung cancer who underwent solid tissue biopsy and cancer-directed therapy at the Cancer Centre of Southeastern Ontario (CCSEO) from 11 February 2022 to 31 May 2023. While tumour biomarker testing in Ontario is available only for non-squamous NSCLC, we included all patients with suspected lung cancer to better align with our project objectives of improving timeliness to biomarker testing and palliative systemic therapy initiation.

We excluded patients who were treated at other institutions, declined treatment, had synchronous malignancies, had a non-lung primary malignancy, or had progression of a previously diagnosed cancer. As part of our parallel control group, we included a cohort of patients with diagnosed lung cancer, who underwent only solid tumour MPS from the same timeline.

### 2.4. Data Collection

We collected data including patient age, sex, Eastern Cooperative Oncology Group (ECOG) performance status, smoking history, lung cancer pathologic subtype and mutation subtype, AJCC 8th edition cancer stage including sites and numbers of metastases, and systemic treatment intent and type, as well as dates of initial LDAP consultation, liquid and solid biopsy, liquid and solid biomarker reporting, medical oncology assessment, and first systemic therapy.

### 2.5. MPS Assay

DNA from collected blood specimens was extracted within 24 h of receipt, using the ThermoFisher MagMAX^TM^ Cell-Free DNA isolation Kit. Assessment of the extracted fragment size profile was performed by a bioanalyzer to look for genomic DNA greater than 500 basepairs in size. When DNA of that size or larger was seen, a magnetic Dynabead purification step was completed to remove the larger molecular weight material. Cell-free DNA was then quantified using the qubit fluorometer and assayed using the Canexia Health Find It^®^/Follow It Assay, with 55 ng of input material, and ligated unique dual indexes for library identification. Libraries were quantified individually, and 20 nM of 13 libraries was pooled, denatured, and loaded at a final concentration of 4 nM using an Illumina MiSeq Reagent Kit v2 300 cycle kit (Illumina, Inc., San Diego, CA, USA).

After sequencing, data were analyzed by the Imagia Canexia Health Insights Platform, using standard analysis algorithms using the bedfile Hotspot Panel: CG001v5.1_Hotspot_Manifest_Panel5.1.3_20210428.tsv. Reporting was performed for single- and multi-nucleotide variants in 37 genes with hotspot coverage. Sequencing encompassed 174 amplicons with an average minimum depth of 1250. Gene amplification events, gene fusion events, and copy number alterations were not in scope.

For economic reasons, ctDNA liquid biopsy analyses were performed in batches of 13, rather than real-time analysis, such that inherent delays were introduced while awaiting sufficient samples to process the batch.

### 2.6. Statistical Analysis

We measured time from solid biopsy/ctDNA blood draw to biomarker reporting and time from solid biopsy/ctDNA blood draw to systemic therapy initiation. Secondary endpoints included time from first clinical assessment to MPS result, time from the ctDNA blood draw to solid biopsy procedure, time from first clinical assessment to palliative systemic therapy, and time from either MPS result to palliative systemic therapy.

Baseline patient, tumour, and treatment characteristics were presented for the solid/liquid biopsy and the solid biopsy-only cohorts. For the primary endpoint (time from solid biopsy/ctDNA blood drawn to systemic therapy initiation), we only included patients with an incurable advanced lung cancer diagnosis, given biomarker-dependent systemic therapy guidance was only indicated in the palliative setting [1,2,3]. We also conducted sensitivity analysis without squamous cell carcinoma histology, given tumour MPS was not routinely indicated in this setting.

During the study period, there were multiple barriers relating to liquid biopsy assay implementation (outlined in Table 1), resulting in intermittent disruptions to patient recruitment. We included patients with conventional solid tumour biopsy only as our parallel control group to enable the contrast of timelines.

All analyses were conducted using IBM SPSS version 29.0 for Windows (Armonk, NY, USA, 2023). Descriptive analyses were used to provide an overview of study population characteristics, including frequencies and percentages for categorical data and means with standard deviations, or medians with quartiles (as appropriate) for continuous data. The underlying distributions of the continuous data were assessed with the Shapiro–Wilk test. No formal analytical comparisons were conducted, and there was no imputation for missing data.

### 2.7. Exploratory Analysis

In a previous 2020 study (unpublished), our local centre had also collected concurrent liquid and solid tumour biopsies using the identical methodology and patient population recruited from the local LDAP, and using the same laboratory staff, equipment, and molecular NGS testing methods, the purpose of which was to assess for feasibility of patient recruitment and concordance between liquid and solid tumour MPS. We conducted an exploratory pooled analysis, including all patients who had both liquid and solid tumour MPS, to increase the sample size for the proportion of concordant results from either source material. Patients who received curative-intent therapy or no oncologic-directed therapy were also included in this exploratory analysis.

## 3. Results

### 3.1. Patient Characteristics

**Figure 2:**
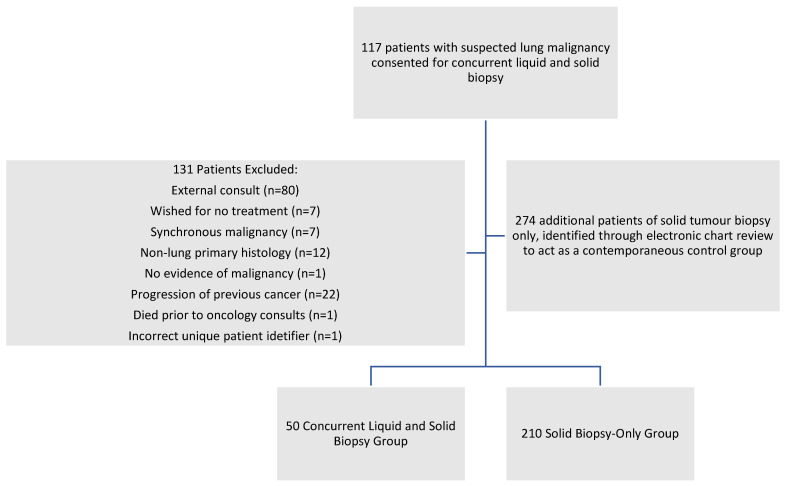
Patient flow diagram.

We identified 117 patients with suspected lung cancer who consented to undergo both liquid and solid tumour biopsy between 1 February 2022 and 31 May 2023. In addition, through retrospective examination of electronic clinical records, we identified 274 patients with confirmed lung malignancy who underwent solid tumour biopsy during the same study period. After accounting for exclusion criteria (*n* = 131, see Figure 2), there were a total of 260 patients in our study, including 50 patients in the concurrent liquid and solid biopsy group and 210 patients in the solid biopsy-only group. Patient demographics, tumours, and systemic therapy characteristics are presented in Table 2.

### 3.2. Timeliness of Care

The median time from date of blood draw to date of liquid biopsy result was 78 days. In the concurrent liquid and solid tumour biopsy group, the median time from date of solid tumour biopsy to biomarker reporting was 22 days, and the median time from date of solid tumour biopsy to palliative systemic therapy was 42 days. The median time from date of liquid biopsy blood draw to palliative systemic therapy initiation was 56 days. For reference, per our solid tumour biopsy-only group, the median times from date of biopsy to MPS sign out and to palliative systemic therapy initiation in all patients were 22 and 47 days, respectively (Table 3).

There were a total of five patients with squamous cell carcinoma histology, two of whom were in the concurrent solid and liquid biopsy group. Similar timeliness patterns were observed within our sensitivity analysis of excluding squamous cell carcinoma histology (Table 3).

There were only four patients whose liquid MPS result was reported earlier than their respective solid tumour MPS results (two cases were 10 days faster, one case was 11 days faster, and one case was 52 days faster). None of these four patients received systemic therapy initiation from the date of plasma MPS results sooner compared to the solid tumour MPS results (*n* = 2 due to receiving palliative radiation therapy first, *n* = 1 due to consensus to repeat solid biopsy, and *n* = 1 due to dental infection delaying systemic therapy initiation).

Clinically, treating medical oncologists used solid tumour biopsy results to guide treatment, as liquid biopsy MPS results came back a median of 19 days after palliative systemic therapy initiation. Solid biopsy MPS results were 39 days faster when compared to liquid MPS results (Table 3), despite liquid biopsy being drawn a median of 11 days earlier than solid biopsy.

### 3.3. Exploratory Analysis of Solid and Liquid MPS Concordance

Of the 50 patients who had both solid and liquid biopsies for MPS, there were 17 targetable mutations identified in the solid tumours (nine cases of KRAS G12C, five cases of EGFR 19 deletion/exon 21 EGFR L858R, and three cases of MET/ROS/ALK fusion). Excluding fusion mutations, which were not analyzed in liquid biopsy MPS, three cases with KRAS G12C and one case with an EGFR activating mutation were not identified by plasma MPS.

Pooled analysis of a total of 85 patients indicated a concordance of 76% in MPS results between the two biopsy specimens (Table 4A). In the discordant samples, there were no mutations detected exclusively in the liquid MPS. KRAS (*n* = 14), TP53 (*n* = 4), MET (*n* = 1), and EGFR (*n* = 1) accounted for the mutations detected in solid MPS that were missed in the analysis of the liquid samples. When only clinically “actionable mutations” were examined, the concordance between two biopsy specimens was 72% (Table 4B).

## 4. Discussion

We sought to evaluate the potential impact of incorporating a liquid biopsy molecular analysis early in the diagnostic pathway for patients with suspected lung cancer. We found that drawing the liquid biopsy at the time of first consultation for suspected lung cancer did not improve the timeliness of first systemic treatment, compared to the routine clinical practice of awaiting tissue biopsy results and MPS. The reasons for this relate primarily to barriers in the timely completion of liquid biopsy analyses, which may be different in other laboratories with various assays and patient volumes. Disruption of global supply chains due to the COVID-19 pandemic impacted our ability to purchase MPS assay components. This development was unexpected but highlighted the need for flexibility in both study design and care pathways for patients. Barriers in implementing liquid biomarkers on turnaround time in our centre had been previously published and, therefore, were not part of this study’s focus [13]. Even though these delays meant that our treating medical oncologists could not use results from liquid biopsies to guide systemic treatment decisions for patients, several lessons warrant further discussions for potential ctDNA future studies.

First, our study highlighted the fact that most MPS assays rely on the use of platforms that are economical only when appropriate batch sizes are available. The need to “right size” the sequencing platform cannot be overlooked when making decisions to add assays to the clinical arsenal. Larger-capacity sequencers, while offering superior costs per base pair of sequencing results, come with the need to have larger numbers of samples or larger assays to fill a run. This capacity must be balanced against the needed turnaround time for individual patients and the flow of samples to the lab. In our case, we used a relatively small platform, but still encountered delays in obtaining sufficient numbers to run batches in a regular cadence. Solutions for this issue include identifying assays that can be co-run on the same flow cell/sequencing chip to increase sample accrual. Alternatively, adoption of liquid biopsy as the standard of care in multiple tumour types would increase sample flow to levels that would remove this barrier. Additionally, incorporating liquid biopsy as a funded diagnostic tool and within the standard of care would increase sample accrual.

A second issue encountered was the failure of ancillary equipment/software during the study. These types of delays are problematic in any laboratory setting and point to the dangers of not having redundant equipment or a second analytic pipeline that can be used when primary approaches fail.

Another potential reason for the lack of observed improvement in timeliness of care with the use of liquid biopsy specimens is the relatively quick turnaround time for patient diagnosis using solid tumour analysis. Biopsies took place, on average, about 11 days after the liquid biopsy was drawn, and NGS results were available 22 days later. In institutions or healthcare environments in which there are more substantial delays in acquiring tissue diagnosis, or perhaps in remote settings, liquid biopsy may have greater potential as a method to improve the timeliness of care. Another potential use of liquid MPS is in circumstances where the solid tumour biopsy is non-diagnostic, or samples are insufficient to process MPS. These non-diagnostic biopsy rates could reach as high as 16.7%, as demonstrated in a retrospective study involving 861 patients who underwent CT-guided percutaneous needle biopsy of the lung [14]. As such, liquid biopsies may be particularly useful in situations where biopsy samples are unable to be processed for biomarkers due to sample size or quality, as well as when there are limitations to obtaining tissue biopsies due to patient comorbidities, technical factors, and/or procedural limitations or availability. Further studies are required to assess the role of liquid biopsy in these settings, particularly when samples are able to be processed in real-time to minimize delays in processing [15].

While study participation was voluntary and we did not collect data regarding the proportion of LDAP patients that were offered or refused liquid biopsy, there were no significant barriers in patient accrual to this study, and it was anecdotally found that patients were receptive to liquid biopsy, particularly when performed at the same time as pre-procedure bloodwork at the time of initial consultation. Results of our ctDNA and solid biomarker reporting also demonstrated high concordance, which was consistent with other reported studies in the NSCLC setting [15,16,17]. In the setting of real-time ctDNA analysis rather than batched analysis, there would be potentially 11 days of improvement in the timeliness of cfDNA blood drawn to biomarker reporting. This is because blood drawn for a liquid biopsy can be obtained on the same day as the initial LDAP consultation, whereas conventional solid tumour biopsies were scheduled on separate days due to required patient preparation and procedure availability. Due to prohibitive costs in our local medium-sized centre, we only conducted liquid biopsy MPS in a batch of 13, thereby contributing to the delay in ctDNA MPS availability. As a comparison, a study of 150 advanced NSCLC patients in a tertiary high-volume cancer centre was able to demonstrate improvement in timeliness of systemic therapy initiation when real-time ctDNA analysis was used for biomarker reporting, when compared to historical solid tumour biopsy biomarker reporting [18]. Another study of 215 patients with suspected advanced lung cancer (ctDNA group = 90 patients) in an academic Ontario cancer centre, demonstrated faster median turnaround time from biopsy to molecular results (14 vs. 35 days, *p* = 0.01) and from medical oncology consultation date to systemic therapy initiation (12 vs. 22 days, *p* = 0.01) for the ctDNA group, when compared to the standard-of-care solid biopsy group [19]. While this paper was also conducted in a similar timeline of 2022 to 2023, the authors did not mention any barriers encountered relating to COVID-19 supply chain disruption, or whether they utilized real-time vs. batch sample liquid biopsy analysis. This study further illustrated our negative results, indicating that a lack of timeliness improvement was mainly driven by local logistical and operational delays rather than by intrinsic limitations of ctDNA technology.

Our study was limited by its relatively small sample size at a single institution. The possibility of enrollment bias exists in that study participation was voluntary, and we did not record the proportion of patients who accepted or declined liquid biopsy. Additionally, the ctDNA biomarker analysis assay was not equipped to detect fusion alterations, albeit these were only detected in 3 of our 50 solid tumour specimens. Lastly, the timeliness of ctDNA to biomarker reporting/systemic therapy initiation was affected by the COVID-19 pandemic disrupting the supply chain; as such, we were not able to reliably utilize cfDNA biomarker reporting to initiate systemic therapy, as the initial protocol intended. Nevertheless, our results demonstrated high concordance rates in ctDNA and solid biopsy biomarkers, warranting future studies that address the identified logistical and operational barriers and target patient populations that are most likely to benefit from timely biomarker testing results to best evaluate the potential benefit of liquid biopsy as a diagnostic tool to improve the timeliness of lung cancer care.

## 5. Conclusions

While our study sought to assess the feasibility of using liquid biopsy as a tool to identify clinically relevant findings in patients with NSCLC, we were not able to demonstrate a positive impact on time to systemic therapy. Identified barriers to success included impediments to the adoption of streamlined testing approaches, including those that were largely unanticipated (impacts of the COVID-19 pandemic) and those that could be solved through more routine adoption of the strategy (the need to batch samples for maximum economic value). While these barriers limited our ability to assess the utility of a liquid biopsy in improving the timeliness of treatment, learnings from our study included demonstrating the feasibility of conducting ctDNA analysis in a small- to mid-sized laboratory, the finding of high biomarker concordance between liquid and solid biopsy, and the relatively high concordance between liquid and tissue biopsy mutations. Further studies that address the identified barriers to implementation are warranted to assess the potential improvement in timeliness of care if liquid biopsy analysis is implemented in real-time.

## Figures and Tables

**Table 1:** Timeline and description of ctDNA implementation barriers.

Time Period	Description of Barrier
11 February 2022	Recruitment of patients for ctDNA in LDAP start date
11 May–31 June 2022	Halted patient recruitment due to supply chain issues limiting ability to analyze liquid biopsy samples
14 June 2022	Supply chain issues resolved; first PCR run of samples from recruited patients
5 July 2022	Recruitment of patients for ctDNA in LDAP resumes
27 July 2022	PCR Run 4, first on-time run, and last sample to make the run received July 26th
14 September–4 October 2022	PCR Run 5 downtime, thermal cycle failure
5 December 2022–23 January 2023	PCR Run 7 downtime, sequencing failure × 2, holiday delay
30 January–6 February 2023	PCR Run 8 downtime, sequencing failure (cracked flow cell)
18 May–24 May 2023	PCR Run 10 downtime, analysis portal failure

PCR = Polymerase chain reaction.

**Table 2:** Baseline patient, tumour, and treatment characteristics.

	Total (N,%)	Concurrent Solid/Liquid Biopsy Group (N, %)	Solid Biopsy-Only Group (N, %)
Age ≥65 years <65 years	196 (75) 64 (25)	30 (60) 20 (40)	166 (79) 44 (21)
Sex Male Female	117 (45) 143 (55)	22 (44) 28 (56)	95 (45) 115 (55)
Type of Biopsy Bronchoscopy/EBUS Non-Bronchoscopy/EBUS	72 (28) 188 (72)	28 (48) 22 (44)	44 (21) 166 (79)
LDAP Involvement Yes No	167 (64) 93 (36)	50 (100) 0 (0)	117 (56) 93 (44)
ECOG 0–1 ≥2	111 (43) 149 (57)	27 (54) 23 (46)	84 (40) 126 (60)
Smoking Status Current Former Never Unknown	96 (37) 136 (52) 25 (10) 3 (1)	19 (38) 24 (48) 6 (12) 1 (2)	77 (37) 112 (53) 19 (9) 2 (1)
Stage 8th Edition I II III IV	48 (18) 20 (8) 41 (16) 151 (58)	0 (0) 0 (0) 6 (12) 44 (88)	48 (23) 20 (10) 35 (17) 107 (50)
Baseline Brain Metastasis Yes No	61 (40) 90 (60)	18 (36) 32 (64)	43 (60) 28 (40)
Number of Baseline Metastases >2 ≤2	95 (63) 56 (37)	14 (28) 36 (72)	81 (80) 20 (20)
Lung Cancer Histology Adenocarcinoma Squamous ** Others	211 (81) 5 (2) 44 (17)	35 (70) 2 (4) 13 (26)	176 (84) 3 (1) 31 (15)
Presence of Actionable Mutations **^++^** Yes No	61 (24) 199 (76)	14 (28) 36 (74)	47 (22) 163 (78)
Systemic Therapy Intent Curative Palliative None	28 (11) 89 (34) 143 (55)	5 (10) 31 (62) 14 (28)	23 (11) 58 (28) 129 (61)
Type of Palliative Systemic Therapy IO Chemotherapy-IO Chemotherapy TT	30 (26) 43 (36) 30 (26) 14 (12)	8 (22) 16 (45) 7 (19) 5 (14)	22 (27) 27 (33) 23 (28) 9 (12)

EBUS = endobronchial ultrasound, LDAP = lung diagnostic assessment program, ECOG = eastern cooperative oncology group, and IO = immunotherapy. ** Others (histology) included large cell (*n* = 8), mixed (*n* = 2), small cell (*n* = 1), and not otherwise specified (*n* = 33). ^++^ actionable mutations included EGFR exon 19 deletion, exon 20 insertion, exon 21 L858R, BRAF V600E/K, and KRAS G12C.

**Table 3:** Timeliness of processes from solid/liquid biopsy to systemic therapy initiation.

	Overall Population		Exclude Squamous Histology	
Time Interval	Solid Tumour Biopsy Only (Median in Days [25th, 75th Percentile])	Concurrent Liquid and Solid Tumour Biopsy (Median in Days [25th, 75th Percentile])	Solid Tumour Biopsy Only (Median in Days [25th, 75th Percentile])	Concurrent Liquid and Solid Tumour Biopsy (Median in Days [25th, 75th Percentile])
Solid Tumour Biopsy to MPS Sign Out	22 (20, 26)	22 (20, 26)	22 {20, 26)	22 (20, 25)
First LDAP/ED Assessment to MPS Sign Out	35 (27, 52)	35 (28, 42)	35 (27, 52)	35 (28, 42)
Solid Tumour Biopsy to Palliative Systemic Therapy	47 (34, 71)	42 (28, 54)	48 (34, 71)	42 (28, 53)
First LDAP/ED Assessment to Palliative Systemic Therapy	62 (46, 90)	58 (44, 76)	62 (46, 92)	58 (45, 76)
Solid Tumour MPS Sign Out to Palliative Systemic Therapy	22 (10, 43)	20 (2, 35)	22 (10, 43)	20 (2, 34)
Liquid Biopsy Draw to Solid Tumour Biopsy	N/A	−11 (−17, −6)	N/A	−11 (−17, −6)
Solid Tumour MPS Sign Out to Liquid Biopsy Result	N/A	39 (10, 82)	N/A	42 (11, 85)
Liquid Biopsy Draw to Liquid Biopsy Result	N/A	78 (49, 129)	N/A	82 (50, 129)
First LDAP/ED Assessment to Plasma Result	N/A	84 (49, 135)	N/A	85 (50, 135)
Liquid Biopsy Draw to Palliative Systemic Therapy	N/A	56 (44, 75)	N/A	56 (45, 76)
Liquid Biopsy Result to Palliative Systemic Therapy	N/A	−19 (−80, −1)	N/A	−23 (−80, −2)

LDAP = lung diagnostic assessment program, and ED = emergency department.

**Table 4:** Exploratory analysis of concordances and discordances between solid and liquid MPS results.

**A. Concordances and Discordances for All Mutations**				
	**Solid MPS Positive**	**Solid MPS Negative**	**Total**	Concordance = 76% Discordance = 24%
Liquid MPS Positive	45	1	46	
Liquid MPS Negative	19	20	39	
Total	64	21	85	
**B. Concordances and Discordances for Actionable Mutations Only**				
	**Solid MPS Positive**	**Solid MPS Negative**	**Total**	Concordance = 72% Discordance = 28%
Liquid MPS Positive	31	1	46	
Liquid MPS Negative	15	11	12	
Total	32	26	58	

MPS = massive parallel sequencing.

## Data Availability

The data presented in this study are available on request from the corresponding author due to (specify the reason for the restriction).
